# Cinnamaldehyde in Focus: Antimicrobial Properties, Biosynthetic Pathway, and Industrial Applications

**DOI:** 10.3390/antibiotics13111095

**Published:** 2024-11-18

**Authors:** Brandon Armando Jaramillo Jimenez, Fatima Awwad, Isabel Desgagné-Penix

**Affiliations:** Department of Chemistry, Biochemistry and Physics, Université du Québec à Trois-Rivières, Trois-Rivières, QC G8Z 4M3, Canada; brandon.armando.jaramillo.jimenez@uqtr.ca (B.A.J.J.); fatima.awwad@usherbrooke.ca (F.A.)

**Keywords:** *trans*-cinnamaldehyde, antimicrobial, specialized metabolism, biosynthesis, aromatic aldehydes, production methods

## Abstract

*Trans*-cinnamaldehyde (TCA), a major bioactive compound derived from cinnamon (*Cinnamomum* spp.), has garnered significant attention for its diverse therapeutic properties. Its broad-spectrum antimicrobial activity, targeting both Gram-positive and Gram-negative bacteria as well as various fungi, positions TCA as a potent natural antimicrobial agent. Beyond its antimicrobial effects, TCA demonstrates promising antidiabetic and anti-inflammatory activities, making it a valuable compound in medicinal and cosmetic applications. Recent studies have highlighted its role in disrupting microbial membranes, inhibiting biofilm formation, and modulating key metabolic pathways in pathogens. Furthermore, TCA has gained popularity in cosmetics due to its antimicrobial activity, antioxidant properties, and skin-friendly profile. This review provides a comprehensive overview of TCA’s antimicrobial potential, focusing on its mechanisms of action and its market and industrial applications. We also discuss the biosynthetic pathway of TCA, exploring both its natural production in cinnamon and advances in biotechnological production methods. As the demand for sustainable and natural antimicrobial agents grows, TCA emerges as a promising candidate for diverse applications. Finally, this review explores future directions for optimizing TCA production through metabolic engineering and synthetic biology approaches to meet industrial-scale demands.

## 1. Introduction

**Figure 1:**
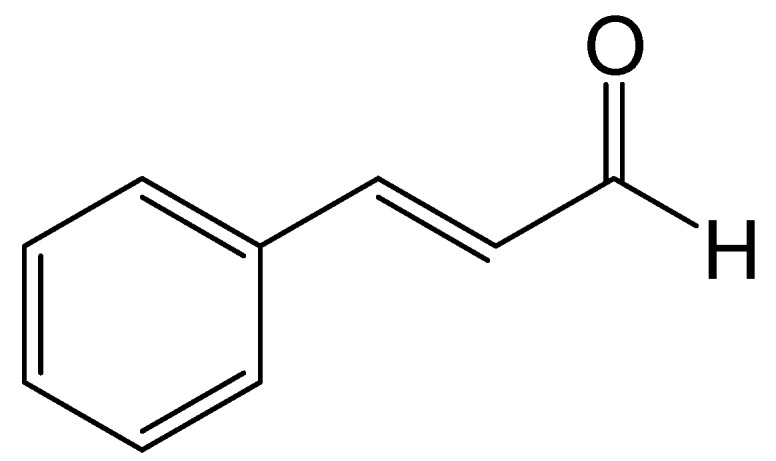
The chemical structure of *trans*-cinnamaldehyde.

*Trans*-cinnamaldehyde (TCA; 3-phenylpro-2-enal; C_9_H_8_O) is an aromatic aldehyde isolated for the first time in 1834 by Dumas and Péligot [1]. It is the primary active compound derived from the bark of cinnamon trees, particularly species of the genus *Cinnamomum*, such as *C. camphora* (camphor) and *C. cassia* (cassia) [2]. TCA is recognized as a naturally occurring antimicrobial compound, and it has been granted Generally Recognized as Safe (GRAS) status by the U.S. Food and Drug Administration [3]. TCA is a yellow, viscous liquid with a strong cinnamon odor and sweet taste, making up 90% of the essential oil of cinnamon. TCA contains two key functional groups: an aldehyde group and a carbon-carbon double bond (Figure 1) [4,5].

Interest in TCA has been increasing due to its applications in various industries, including gastronomy, cosmetics, perfumes, and agriculture. Additionally, it has shown promise in the pharmaceutical sector for its potential role in diabetes prevention [4,6,7]. TCA possesses two electrophilic reactive sites, the first one is the carbon of the aldehyde carbonyl group and the second one is the β-carbon on the conjugated double bond (Figure 1) [8]. Several studies have related the bioactivity of TCA to its ability to act as a Michael acceptor, impairing melanoma cell proliferation, invasiveness, and NF-κB transcriptional activity [9]. Also, recent research has focused on the mechanism of action of TCA and its derivatives, reporting significant antimicrobial activity, which has led to the synthesis of various derivatives of even greater efficacy. Furthermore, it has been shown that the presence of the unsaturated bonded-carbonyl system is essential for biological activities such as inhibiting cell proliferation and mutagenesis [10]. This review will report on the antimicrobial properties of TCA, exploring its mechanisms of action and its potential applications. Additionally, it will discuss its biosynthetic pathway, highlighting key enzymes involved in its natural production, and evaluate recent advances in the biotechnological production of TCA through microbial engineering and synthetic biology approaches aimed at sustainable production.

## 2. Market and Industrial Applications

**Figure 2:**
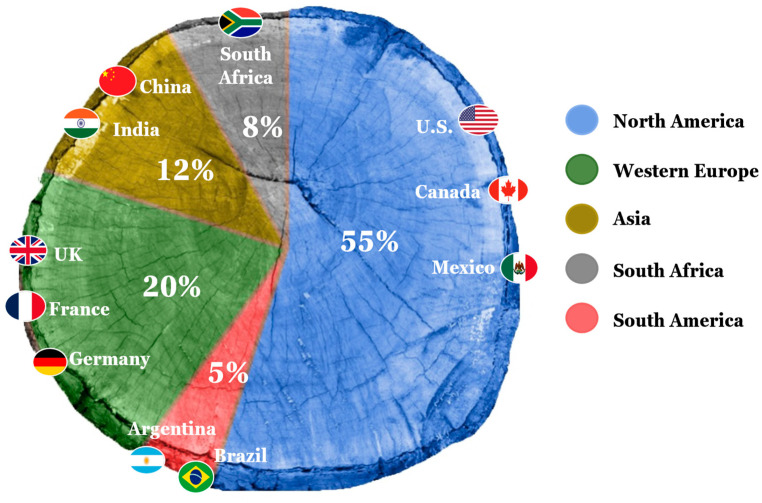
Percentage of global natural cinnamaldehyde market, regional distribution, and expected reach in 2023. North America is the region that leads the TCA industry, followed by Europe and Asia. Adapted from Insights (2023).

The global demand for the natural TCA market has significantly increased recently due to its extensive use in the cosmetics and personal care industries [11]. The global TCA market was valued at 5.3 billion in 2023 and is projected to grow at an annual rate of 4.17% from 2022 to 2032 [12]. North America is expected to be the most lucrative region for natural TCA, offering the greatest market opportunities (Figure 2). However, the global synthetic TCA market has also seen substantial growth in recent years and is anticipated to grow at an annual rate of 6.7% between 2023 and 2033. The Asia-Pacific region is expected to be the most lucrative for synthetic TCA. Key players in the global TCA market include IS Chemical Technology, Labseeker, ABI Chem, Ark Pharma Inc, ApexBio Technology, Timtec, Day Biochem, ChemExper Chemical Directory, Sigma Aldrich, and Bide Pharmatech, among others [12]. Extensive research has been carried out to uncover the various applications associated with TCA, which will be detailed in the following sections.

### 2.1. Antimicrobial Agent

**Figure 3:**
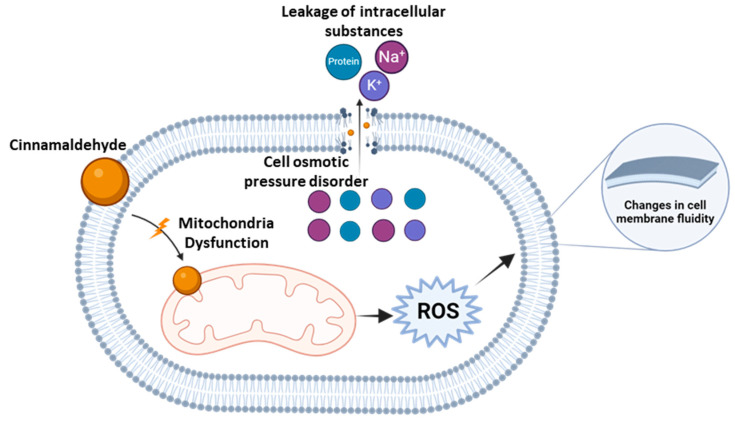
Schematic representation showing the potential inhibition mechanism of *trans*-cinnamaldehyde. TCA changes the cell membrane’s permeability and attacks the mitochondria, leading to the reactive oxygen species (ROS) and leakage of intracellular substances such as Na^+^, K^+^, and proteins. Adapted from [10,20,21]. Created in Biorender.

TCA has demonstrated significant antimicrobial activity and serves as a base for the development of various derivatives [13]. TCA has been shown to effectively inhibit the growth of an array of microorganisms such as bacteria, molds, and yeasts, and it has been reported to inhibit toxin production by microorganisms [14,15]. There have been many studies reporting the mechanism of inactivation of microbial cells by TCA. Here, we summarize some of the most important works. Gill and Holley [16] propose that an interaction with the cell membrane induces rapid inhibition of energy metabolism (Figure 3). The disruption of the proton motive forces results in the leakage of small ions without the leakage of larger components such as ATP, accompanied by the inhibition of ATP generation and membrane-bound adenosine triphosphatase (ATPase) activity. Helander, et al. [17] found that TCA causes a change in the composition of the fatty acids in the membrane, facilitating the cellular incorporation of TCA, which destroys the cell wall and causes the leakage of cytoplasmatic contents such as ions (Na^+^, K^+^, PO_4_^−^), DNA, RNA, and other materials (Figure 3). A study carried out by Amalaradjou and Venkitanarayanan [18] indicated that the mode of antibacterial action of TCA against *Cronobacter sakazakii* was linked to disruptions in amino acid, carbohydrate, and lipid metabolism, which impaired cellular defenses against oxidative stress and reduced *C. sakazakii’s* virulence. Additionally, TCA has been shown to induce reactive oxygen species (ROS) overload and oxidative stress, which disrupts mitochondrial function. It can also trigger apoptosis by causing the release of cytochrome c from the mitochondria into the cytoplasm, thereby increasing ROS levels and activating metacaspases [19].

### 2.2. Antifungal Agent

**Figure 4:**
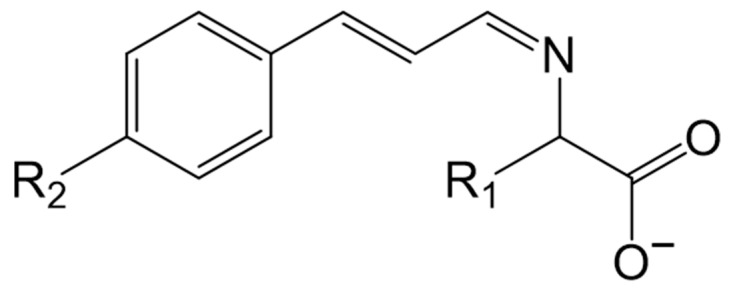
Structure of *trans*-cinnamaldehyde Schiff base compounds.

It has been reported that TCA can act against several pathogenic fungi. It has been shown to exhibit antifungal activity by inhibiting ATPases, disrupting cell wall biosynthesis, and altering the structure and integrity of the fungal membrane [22]. TCA has been shown to inhibit the growth of various fungi through cellular damage and cytoplasmic loss, including species from Eurotiomycetes (*Aspergillus ochraceus*, *Aspergillus flavus*, *Aspergillus fumigatus*, *Trichophyton rubrum*), Sordariomycetes (*Coriolus versicolor*), Trichocomaceae (*Penicillium expansum*), Saccharomycetales (*Saccharomyces cerevisiae*, *Candida albicans*), and *Laetiporus sulphurous* [23]. Furthermore, TCA has been found to reduce spore germination and induce significant changes in the morphology and ultrastructure of fungal hyphae and spores [24]. TCA’s antifungal properties are partly due to the presence of its aldehyde group (–CHO), which can form a Schiff base with amines present in microbial cell components. Furthermore, the addition of a methoxyl group on the TCA benzene ring, along with the development of cinnamaldehyde-amino acid Schiff base compounds using chemical synthesis (Figure 4), enhances some of its antimicrobial, inhibitory, and antifungal properties [25,26].

### 2.3. Antidiabetic Agent

It has been established that TCA plays an important role in managing diabetes in animal models by exhibiting a glycolipid-reducing effect. It enhances glucose absorption, consequently improving insulin sensitivity in adipose and skeletal muscle tissues, thus improving glycogen synthesis in the liver and restoring pancreatic islet dysfunction, decreasing gastric emptying rates, and improving diabetic kidney and brain disorders [6,27]. Extensive studies performed in animal models of diabetes and obesity related to the hypoglycemic and hypolipidemic effects of TCA have demonstrated that oral administration of TCA (20 mg/kg) for a duration from 21 to 60 days resulted in a significant improvement in the levels of blood glucose and glycosylated hemoglobin as well as insulin sensitivity in streptozotocin-induced diabetic rats [4].

### 2.4. Anticancer Activity

It has been demonstrated that one of the most important properties of TCA is its ability to interfere with cancer cell viability. Some studies have shown that the cytotoxic effect of TCA is associated with the increase in ROS and FE as a result of a reversible accumulation of cells in the G2/M cell cycle phases [28]. In addition, it has been reported that the mechanisms through which TCA prevents cancer formation include the induction of apoptosis, interference with cellular invasion and metastasis, etc. They suggest that all these mechanisms are interconnected with one another [29]. Cabello, Bair III, Lamore, Ley, Bause, Azimian, and Wondrak [9] found that TCA suppresses the proliferation of melanoma cells through daily oral ingestion of high doses of TCA, resulting in G_1_ cell cycle arrest and higher intracellular ROS levels. They concluded that TCA induces oxidative stress in A375 cells by up-regulating genes in an expression array.

### 2.5. Cosmetics

TCA is widely used in the cosmetic industry, primarily as a fragrance ingredient in various products. Approximately 95% of its consumption is thanks to its flavoring properties [30]. TCA can be found in fine fragrances, decorative cosmetics, shampoos, and toiletries, as well as in non-cosmetic products like household cleaners and detergents. Globally, TCA is used at an estimated rate of 100 to 1000 metric tons per year [7].

### 2.6. Food Additives

According to the Food and Drug Administration (FDA), and Flavour and Extract Manufacturer’s Association (FEMA), TCA has been Generally Recognized as Safe (GRAS). Moreover, it may be used in foodstuffs thanks to the A status given to it by the Council of Europe [8]. TCA has also become a substitute for synthetic chemical preservatives because it has demonstrated effective antimicrobial activities in food applications [31,32]. TCA imparts a cinnamon flavor to foods and is also used as an antioxidant and a natural food preservative in products such as drinks, candies, ice cream, chewing gum, and condiments to protect against fungi (Table 1) [33,34]. Approximately 180,000 kg of TCA are consumed annually in foods, of which 39,000 kg are consumed via the use of cinnamon and 141,000 kg are due to its deliberate addition as a flavor [24].

## 3. General Metabolic Pathway to Produce Cinnamaldehyde

Plants can produce natural organic compounds derived from a specialized metabolism that does not directly impact their growth and development [41]. The most important active compounds originate from different precursors of their primary metabolism and are synthesized through separate metabolic pathways. These compounds are divided into chemical groups such as alkaloids, terpenoids, and phenolic compounds [42]. The phenolic compounds are a diverse group widely distributed in plants and divided into several sub-groups based on their structural complexity, including phenylpropanoids, flavonoids, tannins, and lignans [43]. While phenylpropanoids are typically not found in essential oils (usually terpenoids), some plants, such as *C. cassia*, contain them in significant proportions. In fact, TCA is the most abundant of the major phenylpropanoids produced. It can be obtained from the plant’s volatile fraction via steam distillation [44].

**Figure 5:**
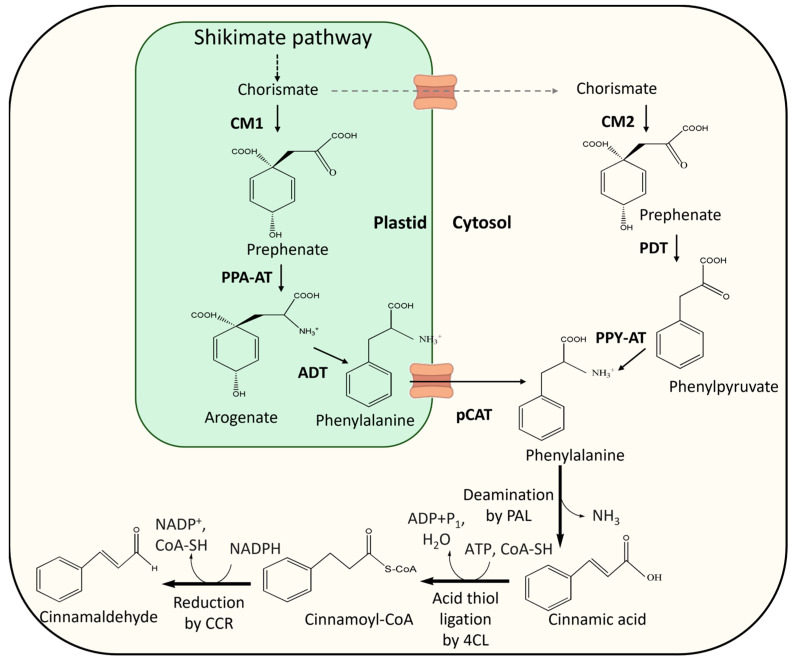
Proposed cinnamaldehyde biosynthetic pathway in microalgae. Black dotted lines show multiple enzymatic steps involved. Black arrows represent enzymatic, or transport steps supported by direct experimental evidence. Gray dashed arrows represent hypothesized steps. Enzyme abbreviations: CM, chorismate mutase; PPA-AT, prephenate aminotransferase; ADT, arogenate dehydratase; pCAT, plastidial cationic amino acid transporter; PDT, prephenate dehydratase; PPY-AT, phenylpyruvate aminotransferase; PAL, phenylalanine ammonia-lyase 4CL, 4-coumarate-CoA ligase; CCR, cinnamoyl CoA reductase. Adapted from [48,49,50,51,52]). Created in ChemDraw.

Phenylpropanoids are mainly derived from the aromatic amino acid precursor phenylalanine, which is synthesized through the shikimate metabolic pathway, a metabolic route functional only in microorganisms and plants (Figure 5) [45,46]. Although the phenylpropanoid pathway is not yet fully characterized, the most widely accepted model for the biosynthesis of TCA in plants involves three enzymatic reactions. First, the non-oxidative deamination of phenylalanine into cinnamic acid by phenylalanine-ammonia lyase (PAL). Next, cinnamic acid undergoes acid–thiol ligation with CoA, forming cinnamoyl CoA through the action of 4-coumarate: CoA ligase (4CL). Finally, cinnamoyl-CoA is reduced to TCA by cinnamoyl-CoA reductase (CCR) [43,47,48].

### 3.1. Precursor Biosynthesis: Chorismate and Phenylalanine

The shikimate pathway is responsible for the production of chorismate, which is a common precursor for the biosynthesis of aromatic amino acids (tyrosine, tryptophane, and phenylalanine) [43]. At the same time, phenylalanine is required as an essential precursor for the biosynthesis of TCA [48]. Given the importance of the shikimate pathway, all members of the biosynthetic genes and corresponding enzymes have been identified and characterized in model plants such as *Arabidopsis* due to their homology genes from microbial organisms [53]. In addition, a cross-species comparison of the shikimate enzymes was carried out, and it revealed that they exhibit similarities in their sequences, have undergone divergent evolution, and share commonalities in reaction mechanisms. [43]. However, the shikimate pathway genes in plants stem from at least three distinct sources and do not originate from a single prokaryotic ancestor of cyanobacterial origin [54]. During the evolutionary development of this pathway, there was a complex process involving multiple steps of functional loss and gain, which could elucidate the various factors influencing the genomic organization and expression of pathway genes in plants [55]. The shikimate pathway exists exclusively in plants and microorganisms, and this originates from precursors of the glycolysis and phosphate pentose pathways. It undergoes seven enzymatic reactions [56]. The synthesis of chorismate is a crucial step in the shikimate pathway and begins with the condensation of erythrose 4-phosphate and phosphoenolpyruvate. This reaction is catalyzed by 2-dehydro-3-deoxyphosphoheptonate aldolase and results in 2-dehydro-3-deoxyphosphoheptonate, which is used to form 3-dehydroquinate in a reaction of cyclization catalyzed by 3-dehydroquinate synthase. Then, 3-dehydroquinate dehydratase converts 3-dehydoquinate into 3-dehydroshikimate, which is reduced to shikimate in a reaction catalyzed by a shikimate-NADP oxidoreductase. Consequently, there is a phosphorylation of shikimate to form shikimate-3-phosphate. The reaction is catalyzed by shikimate kinase. The next reaction is catalyzed by 5-enolpyruvylshikimate-3-phosphate synthase, which is used to produce 5-enolpyruvylshikimate-3-phosphate. Finally, the last step in the synthesis of chorismate is carried out by chorismate synthase, converting 5-enolpyruvylshikimate-3-phosphate into chorismate [57]. The next intermediate in the synthesis pathway of phenylalanine is catalyzed by the action of chorismate mutase (Figure 5), which converts chorismate into prephenate. Finally, based on the identified genes, there may be two branching points in the production of phenylalanine, either due to the action of phenylpyruvate or due to arogenate dehydratase, which catalyze the final steps for the production of phenylalanine [57,58].

### 3.2. Enzymes Involved in the Production of Cinnamaldehyde

#### 3.2.1. Phenylalanine Ammonia-Lyase (EC 4.3.1.24, PAL)

Phenylalanine ammonia-lyase (PAL) is a ubiquitous enzyme that can be isolated and characterized from plants, fungi, and some bacteria, and it fosters diverse activities and specificities according to the origin of the enzyme [59]. PAL catalyzes the non-oxidative deamination of phenylalanine to *trans*-cinnamic acid. A carbon-carbon double bond is formed during the release of NH_3_ (Figure 5), yielding *trans*-cinnamic acid. This reaction is considered as a key entry and is the first step of the phenylpropanoid pathway [60]. The activity of PAL can be induced in response to biotic and abiotic stresses. Moreover, due to its importance in phenylpropanoid metabolism, PAL has been purified from different organisms (Table 2), most of them from plant species such as *Arabidopsis thaliana* (four genes), *Populus trichocarpa* (five genes), *Scutellaria baicalensis* (three genes), *Cucumis sativus* (seven genes), and *Coffea canephora* (three genes) [61].

#### 3.2.2. 4-Coumarate-CoA Ligase (EC 6.2.1.12, 4CL)

4-coumarate-CoA ligase (4CL) participates in the reaction to convert *trans*-cinnamic acid to *trans*-cinnamoyl-CoA by an acid–thiol ligation biosynthesis using ATP (Figure 4) [48]. 4CL is the main branch point in the phenylpropanoid pathway, and it is required for the biosynthesis of precursors for different phenylpropanoids in plants such as lignin, phenylpropanoid esters, phenolic glycosides, flavonoids, and soluble metabolites [62]. Owing to its role as the main branch point, 4CL can convert cinnamic acid derivatives, including p-coumaric, caffeic, ferulic, and cinnamic acids, into a distinct coenzyme A (CoA) thioester [63]. Several genetic screens have been carried out to identify 4CL in different plants (Table 1) such as *Populus tomentosa*, *Glycine max*, and (with a special focus) in *Arabidopsis thaliana*, where 4CL is encoded by four genes (*4CL1*, *4CL2*, *4CL3*, and *4CL4*). The first three genes have been shown to use p-coumaric acid, caffeic acid, and ferulic acid as substrates. Only *4CL4* is capable of activating sinapic acid [64,65,66].

#### 3.2.3. Cinnamoyl CoA Reductase (EC 1.2.1.44, CCR)

Cinnamoyl CoA reductase (CCR) participates in the reaction in which *trans*-cinnamoyl-CoA is reduced by NADPH catalyzed by the cinnamoyl-CoA reductase (CCR) to form *trans*-cinnamaldehyde (Figure 4) [48]. CCR is also considered the first enzyme in the monolignol-specific branch of the phenylpropanoid pathway, and it catalyzes the conversion of cinnamoyl CoA esters to their corresponding cinnamaldehydes, i.e., the first specific step in the synthesis of the lignin monomers [67]. In addition, most of the studies on CCR are related to the lignin-specific pathway and how CCR plays a role in the lignin biosynthesis pathway, due to this enzyme occupying a key position between the general phenylpropanoid pathway and the lignin-specific branch regulating the carbon flux toward lignins by gene transfer experiments [68,69]. CCR has been successfully cloned from *C. cassia* (*CcCCR1*) and *Arabidopsis thaliana* (*AtCCR1*) (Table 2), and higher catalytic efficiency was found in *CcCCR1*, showing superior catalytic activity [70].

**Table 2:** Identification and kinetic parameters (*K_m_ =* Michaelis constant; *k*_cat_ *=* catalytic constant) of phenylalanine ammonia-lyase (PAL), 4-coumarate–CoA ligase (4CL), and Cinnamoyl-CoA reductase (CCR) genes in plant species.

Substrate	Organism	Gene	*K_m_* (μM)	*k*_cat_ (S^−1^)	Reference
Phenylalanine	*Arabidopsis thaliana*	*PAL1*	658 ± 47	11.9	[71]
Phenylalanine	*Arabidopsis thaliana*	*PAL1*	68 ± 5	1.8	[72]
		*PAL2*	64 ± 3.5	3.2	
		*PAL3*	2560 ± 340	0.1	
		*PAL4*	71 ± 3	3.0	
Phenylalanine	*Populus trichocarpa*	*PAL1*	81.6 ± 0.30	22.79	[73]
		*PAL2*	32.35 ± 0.50	8.35	
4-Coumarate	*Arabidopsis thaliana*	*4CL1*	38	-	[74]
		*4CL2*	252	-	
		*4CL3*	23	-	
4-Coumarate	*Populus tomentosa*	*4CL*	6.79 ± 3.53	4.24	[75]
4-Coumarate	*Glycine max*	*4CL1*	22 ± 32	-	[76]
		*4CL2*	42 ± 17	-	
Coumaroyl-CoA	*Arabidopsis thaliana*	*CCR1*	2.27 ± 0.07	1.63	[77]

## 4. Production with Traditional and Novel Methods

The traditional methods of TCA production are the cultivation of and chemical synthesis, such as aldol condensation of benzaldehyde and acetaldehyde and oxidative or catalytic processes [11]. The essential oil from Cinnamomum tree bark is rich in *trans*-cinnamaldehyde, which has antimicrobial effects against animal and plant pathogens, food poisoning, and spoilage bacteria and fungi [78]. The essential oil from the bark of *C. cassia* and *C. verum* contains varying concentrations of TCA, typically ranging between 1% and 12% in most samples [79,80,81]. However, the oil itself can be enriched to have TCA as the major constituent, sometimes reaching up to 85% in *C. cassia* and 90% in *C. verum* oils, depending on the extraction methods and quality [82,83]. The bark and leaves of *Cinnamomum* spp. are commonly used as spices in home kitchens, and their distilled essential oils are used as flavoring agents in the food [84] and beverage industries [85]. In addition, an alternative method of producing TCA is chemical synthesis through the condensation of benzaldehyde and acetaldehyde. High selectivity and conversion can be achieved using chemical synthesis, facilitating the separation of products [11]. Different methods exist to extract bioactive components from plant matrices, such as pressure drop fractional distillation, water steam distillation, Soxhlet extraction, reflux extraction, supercritical fluid extraction, etc. All of these methods enhance purity and recovery [86]. One of the most used methods is water steam distillation. The cinnamon oil is mixed with dichloromethane and dried with sodium sulfate, followed by evaporation using a rotary evaporator. The isolation of TCA is obtained after 6 h [87]. Furthermore, another famous method of extracting TCA is subcritical water extraction, using high pressures and water at a high temperature of around 100–375 °C, which allows water to act like an organic solvent [88]. Finally, supercritical fluid extraction employs CO_2_ to act as a powerful solvent. The principle of this method combines liquid-like density and solvation power with gas-like diffusion and low viscosity, where distinct gas and liquid phases disappear. This method achieves a high extraction efficiency [89]. However, there are disadvantages to these production and extraction methods, such as the thermal degradation that can occur over a prolonged extraction time at high temperatures, and a longer extraction time requires a large amount of solvent and leads to the degradation of bioactive compounds [2,48]. Additionally, the systematic cultivation process requires up to two to three years for the plant to grow completely, which is why it is not sustainable or economically feasible in either the short or the long term [11,90].

Nevertheless, in recent years, a few research studies have been carried out to develop alternative strategies focused on reaching high-efficiency TCA production by using microorganisms such as bacteria and yeast as cell factories. Bang, Lee, Kim, Sung, and Jeong [48], performed metabolic engineering in *Escherichia coli* by constructing the biosynthesis pathway to produce TCA. Three biosynthetic enzymes, phenylalanine-ammonia lyase (PAL), 4-coumarate: CoA ligase (4CL), and cinnamoyl-CoA reductase (CCR), were cloned into pTrc99A vectors corresponding to the bacteria *Streptomyces maritimus*, *S. coelicolor*, and *A. thaliana* respectively. The expression of the three synthesis genes was carried out under the IPTG-inducible trc promoter (Ptrc). Finally, *E. coli* W3110 was cultivated harboring pHB-TCA (yielding *SmPAL*, *ScCCL*, and *AtCCR*). Moreover, *E. coli* was modified to increase the intracellular pool of phenylalanine, which is the main precursor of TCA. According to this result, TCA production rates as high as 75 mg/L could be achieved. Despite the successes of the transformation, a new study was carried out by Bang, et al. [91], this time using systematic metabolic engineering. In this study, the H-02 strain was engineered. First to be constructed was an expression system to overproduce phenylalanine through the efficient conversion of *trans*-cinnamic acid to TCA. In addition, the deletion of ten endogenous genes reduced the loss of TCA to cinnamyl alcohol. Next, an auto-inducible promoter was used instead of an IPTG-inducible promoter, and the expression systems for TCA biosynthesis were integrated into chromosomal DNA. Subsequently, acetate pathways were deleted and pools of available cofactors were increased to facilitate TCA production. Finally, fed-batch cultivation with in situ product recovery was performed, achieving a production titer of TCA as high as 3.8 g/L, which is the highest TCA titer ever reported. Another similar study was carried out by Son, et al. [92]. They engineered *Corynebacterium glutamicum* as a whole-cell biocatalyst for the efficient bioconversion of *trans*-cinnamic acid (*t*-CA) into TCA. To prevent the loss of TCA, the putative dehydrogenase-related genes (*dkgA*, *adhC*, and *cg*1176) were deleted. In addition, the replacement of the putative promoter region of the *zwf* gene with a strong promoter was achieved. Finally, the deletion of the *vdh* gene was involved in the reverse conversion of TCA to *t*-CA. As a result, a 100% conversion yield of 1.1 g/L TCA from 1.2 g/L *t*-CA was obtained within 30 min. Nevertheless, the production titer was not sufficient for commercialization. This is why additional improvements are still required to increase and improve enzymatic reactions, thus allowing significantly higher production titers. Finally, Gottardi, et al. [93] engineered the yeast *Saccharomyces cerevisiae* to produce *trans*-cinnamic acid derivatives such as TCA from externally added *trans*-cinnamic acid. The study showed that *S. cerevisiae,* through the overexpression of genes such as phenylalanine ammonia lyase 2 from *A. thaliana* (*AtPAL2*), aryl carboxylic acid reductase (acar) from *Nocardia* sp., and phosphopantetheinyl transferase (entD) from *E. coli*, is able to produce TCA, but the strains are not capable of tolerating high concentrations.

## 5. Metabolic Engineering Strategies in Microbes for the Production of Cinnamaldehyde

Previous studies have been focused on providing a proof of concept and a strain that can be further optimized to be capable of synthesizing high-value compounds such as TCA [94]. It is therefore important that a background strain suitable for phenylpropanoid production should be elaborated first, either by increasing the expression level of the shikimate and chorismate pathways or by making an inducible expression of CM, PAT, or ADT (Figure 4). The toxicity of intermediates in a pathway can affect the production of end products such as TCA. A study carried out by Gottardi, et al. [93] showed that TCA toxicity in yeast cells affects their growth, suggesting that the production of TCA derivatives is likely to pose other challenges beyond establishing a functional heterologous pathway. It is therefore crucial to have a temporally accurate gene regulatory system when over- or under-expression of the target gene may be fatal to the host cell. Therefore, the ability to modulate gene expression through an inducible expression system becomes critical [95]. These factors highlight the need for the development of a sustainable microbial-based production system through steps of rational metabolic engineering and also de novo synthesis via the heterologous overexpression of different genes [91,93]. Nevertheless, in recent years, a significant challenge has emerged in developing microorganism chassis for biomanufacturing while also aligning with the biorefinery concept to generate economically viable solutions. These challenges have spurred the development and application of several synthetic biology tools across different microorganisms, enabling the construction of more controllable, standardized, and predictable biotechnological chassis, achieving these goals in less time and at reduced costs [96,97]. The main next-generation engineering strategies include gene knockout tools, targeted genetic engineering via transcription activator-like effector nucleases (TALENs), CRISPR-Cas9 for gene-targeted mutations, extrachromosomal transgene expression via independent episomes, chloroplast transformation systems, and large episomes that contain various DNA parts that can be maintained and expressed without requiring genomic integration [96,98,99,100,101]. Moreover, another viable solution is the availability of bacterial conjugation for economic genetic transformations, leaving behind the more expensive alternatives such as biolistic and electroporation [102]. In this mechanism, the DNA is replicated stably within the nucleus as an episome, introducing a huge prospect for artificial chromosome transfer into diatoms, resulting in a one-step transfer of whole metabolic pathways [97]. All of these genome editing tools provide the ability to transform organisms into chassis capable of producing non-native components that are not naturally found in the organism [103].

## 6. Conclusions

In this review, we have presented the different properties of TCA, highlighting its antimicrobial properties as well as its industrial applications. Furthermore, advances in metabolic engineering have made it possible to replicate TCA biosynthesis in non-native organisms such as bacteria, opening up new possibilities for the sustainable production of TCA in biotechnological systems. In the industrial field, TCA has proven to be essential not only to the formulation of flavors and fragrances but also as a precursor in the synthesis of compounds of commercial interest, as well as in its application in agriculture as a natural biopesticide and biofertilizer. These advances, together with its optimized production through bioprocesses, underline the enormous potential of TCA as a versatile and high-value molecule in the search for more sustainable and efficient industrial solutions.

## Figures and Tables

**Table 1:** Usage of cinnamaldehyde as a preservative in food against different organisms.

Organism	Target	Reference
*Aspergillus parasiticus*	Maize grain	[35]
*Aspergillus flavus*	Corn	[19]
*Aspergillus niger*	Bread	[23]
* Escherichia coli* & *Staphylococcus aureus *	Cosmetics	[36]
* Enterobacteriaceae *	Chicken liver	[37]
* Penicillium citrinum *	Bamboo	[38]
* Fusarium verticillioides *	Grain	[39]
* Penicillium* spp. & *Aspergillus * spp.	Baked foods	[40]

## Data Availability

The data are available on request from the corresponding author.
